# Positioning Transclival Tumor-Treating Fields for the Treatment of Diffuse Intrinsic Pontine Gliomas

**DOI:** 10.3390/life13030601

**Published:** 2023-02-21

**Authors:** Walid Ibn Essayed, Casey A. Jarvis, Joshua D. Bernstock, Anna Slingerland, John Albanese, Gregory K. Friedman, Omar Arnaout, Lissa Baird

**Affiliations:** 1 Department of Neurosurgery, Brigham and Women’s Hospital, Harvard Medical School, Boston, MA 02115, USA; 2 Department of Neurosurgery, Boston Children’s Hospital, Harvard Medical School, Boston, MA 02144, USA; 3 David H. Koch Institute for Integrative Cancer Research, Massachusetts Institute of Technology, Cambridge, MA 02139, USA; 4 Department of Pediatrics, Division of Pediatric Hematology and Oncology, University of Alabama at Birmingham, Birmingham, AL 35294, USA

**Keywords:** clivus, diffuse intrinsic pontine glioma (DIPG), endoscopic endonasal surgery, intratumoral modulation therapy, tumor-treating fields (TTF)

## Abstract

Diffuse intrinsic pontine glioma (DIPG) carries an extremely poor prognosis, with 2-year survival rates of <10% despite the maximal radiation therapy. DIPG cells have previously been shown to be sensitive to low-intensity electric fields in vitro. Accordingly, we sought to determine if the endoscopic endonasal (EE) implantation of an electrode array in the clivus would be feasible for the application of tumor-treating fields (TTF) in DIPG. Anatomic constraints are the main limitation in pediatric EE approaches. In our Boston Children’s Hospital’s DIPG cohort, we measured the average intercarotid distance (1.68 ± 0.36 cm), clival width (1.62 ± 0.19 cm), and clival length from the base of the sella (1.43 ± 0.69 cm). Using a linear regression model, we found that only clival length and sphenoid pneumatization were significantly associated with age (R^2^ = 0.568, *p* = 0.005 *; R^2^ = 0.605, *p* = 0.0002 *). Critically, neither of these parameters represent limitations to the implantation of a device within the dimensions of those currently available. Our findings confirm that the anatomy present within this age group is amenable to the placement of a 2 × 1 cm electrode array in 94% of patients examined. Our work serves to demonstrate the feasibility of implantable transclival devices for the provision of TTFs as a novel adjunctive therapy for DIPG.

## 1. Introduction

Diffuse intrinsic pontine gliomas (DIPG), a subset of diffuse midline gliomas that are *H3K27* altered, as classified by the fifth edition of the WHO’s Classification of Tumors of the Central Nervous System (CNS), are highly fatal pediatric brainstem tumors for which there are no curative treatment options [1,2]. As such, the prognosis remains dismal, with a 2-year survival rate of <10% despite maximal radiotherapy [1,3]. A litany of experimental therapeutics is being trialed for DIPG, with some encouraging early results having recently emerged from clinical trials utilizing adoptive cell transfer and immunovirotherapy [4,5,6,7,8].

An emerging treatment for high-grade gliomas is electrotherapy, a unique modality in which tumor cells are exposed to low-intensity (1–3 V) intermediate frequency (100–500 kHz) electric fields capable of inhibiting tumor cell growth [9,10]. There are multiple described applications of intermediate frequency electrotherapy, including pulsed electric fields, which induce electroporation, and tumor-treating fields (TTF) [9]. TTF are alternating current electric fields which are created by externally fixed arrays on the scalp, the arrangement of which can be individualized [11,12]. The mechanism of action remains controversial; however, the prevailing hypothesis is the disruption of mitosis in dividing malignant cells [10,11]. Intratumoral modulation therapy (IMT) is a derivative approach of TTF which involves the local delivery of low-intensity sinusoidal intermediate-frequency electric fields by means of implanting field-generating electrodes within targeted tissue [12,13]. A report by Deweyert et al. attempted to evaluate the potential role of this intratumoral modulation therapy in DIPG via an in vitro pilot study [14]. The results demonstrated marked susceptibility of DIPG cells to low-intensity electric fields, with a significant impact both as mono- and multimodal therapy, thus warranting further investigation [14].

It is, however, prudent to note that the intra-axial implantation of electrodes is associated with a number of non-negligible risks, the foremost of which is an injury to surrounding eloquent structures, particularly in highly functional tissue such as the brainstem [15,16]. In addition, permanently implanted intra-axial hardware poses its own associated limitations and risks, including infection, migration, and/or dysfunction, any of which may require revision surgeries and prolonged hospital stays [15,17]. With current electrode technology, tissue coverage achieved by an IMT electrode is limited, which also would require the placement of multiple electrodes to cover larger lesions [14]. Finally, another critical constraint of an internally implanted system is the question of MRI compatibility, a crucial modality in the management/monitoring of patients with brain tumors.

Due to such limitations, the current clinical usage of tumor-treating fields (TTF) in CNS cancers has been confined to external systems, such as the transcranial scalp electrode system commercially available by Optune, in the management of glioblastoma (GBM) [11,12]. The results of TTF in GBM are encouraging, with a 2.7-month and 4.9-month increase in progression-free survival (PFS) and overall survival (OS) observed, respectively, when used in combination with current therapeutic standards [11,12]. However, one of the inherent limitations in the setting of transcranial field generation is the depth of targeted tissue and the insulating effect of the skull [18,19]. Clinical evidence has demonstrated that field intensity delivered to the site of the tumor is correlated with overall survival [18,19,20]. Accordingly, one active area of research in TTF is cranial remodeling interventions, in which the calvarial bone is either surgically thinned and/or burr holes or small craniectomies are strategically placed either during the initial tumor resection or re-resections with the aim of enhancing TTF penetrance and intensity [20,21,22].

To our knowledge, there is no published data regarding the treatment of DIPG patients with TTF due in part to the various anatomical and engineering challenges defined above. Given encouraging in vitro data and the need for focused, non-toxic pediatric therapies, a deeper investigation of this modality is warranted. As such, we sought to clarify the practicability of placing an implantable array in close proximity to the pons using a transnasal corridor. This concept involves a hybrid approach taking advantage of the anatomical location of DIPG and is centered on the implantation of an intracranial extra-dural electrode embedded within the clivus, just ventral to the pons. To accomplish this proof-of-concept study, we evaluated the anatomical feasibility of endoscopic endonasal placement of TTF electrodes in pediatric DIPG. The feasibility of this approach represents an integral step in the process of developing a clival electrode form factor and investigating clival TTF as a novel treatment method for DIPG, with the ultimate goal of further evaluating this technology in a clinical setting.

## 2. Materials and Methods

We retrospectively reviewed patients from our institution who presented with a clinical, radiographic, and histologically confirmed diagnosis of DIPG, from 2010 to 2022. An algorithm was used to interrogate our institutional database using the keywords “diffuse intrinsic pontine glioma” or “DIPG” to identify patient charts. These charts were then manually reviewed to verify the diagnosis of DIPG and the presence of CT scans. Patients who did not have a CT scan were excluded. This protocol was reviewed by the local IRB under protocol number IRB-P00027869 and has an approved waiver of consent to conduct retrospective research.

**Figure 1:**
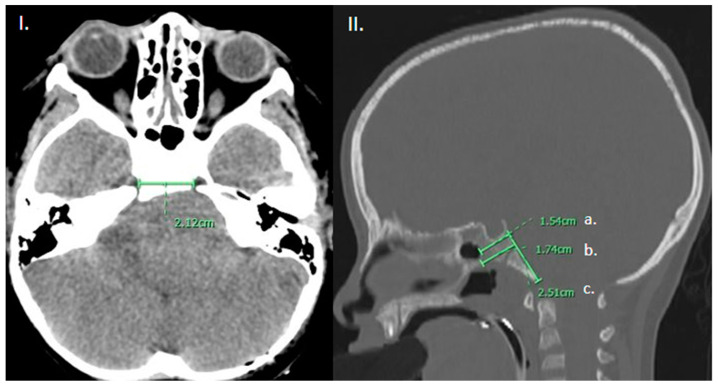
(**I**). Axial CT brain demonstrating measurement of the intercarotid distance. (**II**). Sagittal CT head, bone window in the mid-sagittal plane demonstrating measurement of the (a) thickness of the clivus at the level of the sellar floor as well as (b) at the thickest portion measurable and (c) height of the clivus from sellar floor to basion.

We characterized the anatomical features that limit transclival approaches. The width of the approach was measured as the minimum lacerum inter-carotid distance. The degree of pneumatization of the sphenoidal sinus was divided into conchal, pre-sellar, sellar, and post-sellar, as has been previously described in the literature [23]. The height of the clivus was measured in the midsagittal plane extending from the base of the sella to the basion. The thickness of the clivus was measured at two levels: at the level of the sellar floor and at the thickest portion measurable on the midsagittal reconstruction (Figure 1).

Linear regression analyses were conducted to determine the relationships between age at the time of the CT and all anatomical measurements, including sphenoid pneumatization. The degree of pneumatization was then codified from 1–4 with 1 representing conchal, 2 pre-sellar, 3 sellar, and 4 post-sellar, for logistic regression analysis. All statistical analyses were conducted using StatPlus. A *p*-value of <0.05 was used to determine significance. The figures were created using BioRender.com or 3D slicer.

## 3. Results

Forty-three unique patients were algorithmically identified as having a diagnosis of DIPG. Of these 43 patients, 17 patients had a verified tissue diagnosis of DIPG, and also had CT scans with sagittal/axial sequences of appropriate quality to complete the desired measurements. Of these patients, ten were male and seven were female. Ages ranged from 4–15 years; the median age was 6 years. Of sphenoid pneumatization categories, six patients fell within the conchal pneumatization type, five were pre-sellar, four were sellar, and one was a post-sellar type (Table 1). The average height of the clivus from the base of the sella to the basion was 3.34 ± 0.39 cm. The thickness of the clivus from the base of the sella was on average 1.43 ± 0.69 cm. The greatest thickness of the clivus was on average 1.62 ± 0.19 cm. The average inter-carotid distance was 1.67 ± 0.36 cm (Table 2). Age at the time of CT was found to be a statistically significant predictor of clival length (R^2^ = 0.568, *p* = 0.0005), the width of clivus at the sellar floor (R^2^ = 0.372, *p* = 0.009), and degree of sphenoid pneumatization (R^2^ = 0.605, *p* = 0.0002). Age was not a significant predictor of the greatest width of clivus (R^2^ = 0.004, *p* = 0.82), or inter-carotid distance ((R^2^ = 0.006, *p* = 0.76) (Table 3).

## 4. Discussion

TTFs represent a non-invasive biophysical approach to the management of different cancers, including pancreatic, ovarian, lung, and brain tumors, and have become a fourth treatment modality alongside surgery, chemotherapy, and radiation [11]. TTF for GBM involves applying alternating electric fields at intermediate frequencies and requires that the therapy be performed for greater than 18 h per day in order to confer clinical benefit [11,24]. Multiple biomolecular mechanisms are involved in the antineoplastic effects of TTF [25]. The antimitotic effect is the most widely accepted mechanism, yet a myriad of other means have also been hypothesized and tested, including DNA-damage response, suppression of cancer cell migration, autophagy, innate immunity, and immunogenic cell death [11].

**Figure 2:**
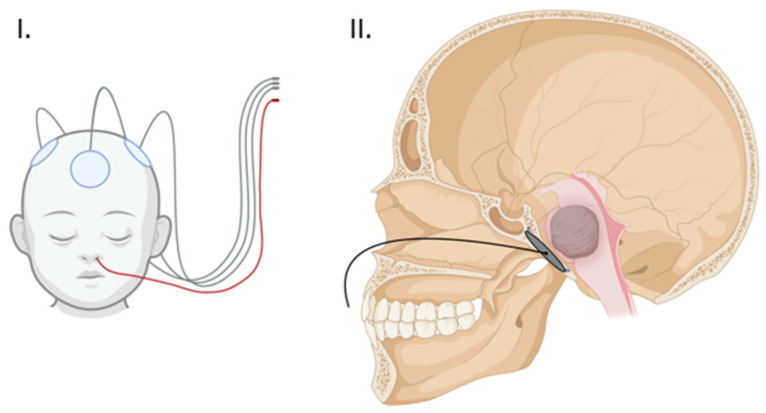
(**I**) Proposed external array configuration demonstrating the clival array wiring exiting the nose in red. (**II**) Sagittal view of the clival array placement with transnasal wiring. (Created with BioRender.com).

One of the most salient limitations of TTFs is the physical barrier of the surrounding soft tissue and skull which can lead to a significant dampening of field intensity, thereby decreasing treatment efficacy [18,26]. Experimental modeling has shown the potential improvement of TTF efficacy by decreasing skull thickness through the strategic use of surgical thinning or bone-removal techniques [18]. A recent phase I trial of Skull Remodeling Surgery (SR-Surgery) performed during the resection of the GBM demonstrated the safety of utilizing calvarial-thinning techniques along with multiple burr holes in and around the bone flap. A phase II trial is currently ongoing with the aim of confirming the efficacy of this approach [20]. These encouraging results suggest the utility of similar approaches in the skull base to access and therapeutically engage deeply located lesions [18,22]. Our study sought to employ these surgical remodeling techniques in the skull base and take advantage of the anatomical location of DIPG in the pons, just ventral to the clivus, via endoscopic implantation of an external TTF array within a surgically thinned clivus (Figure 2).

DIPG cells have been shown to be sensitive to low-frequency electrical stimulation in vitro as evidenced by reduced cell viability and increased apoptosis [14]. When administered in combination with temozolomide (TMZ) and radiation therapy (RT), IMT combination therapy is synergistic, achieving better results than monotherapy or dual TMZ-RT [14]. Intratumoral or peritumoral electrode placement is limited in this application due to the high degree of eloquence within the brainstem [12]. Given the diffuse nature of pontine gliomas, it would be difficult to safely place enough electrodes in the parenchyma to provide sufficient coverage and produce a potential clinical benefit. TTF, which does not necessitate the placement in the parenchyma, seem to be a preferable option for eloquent brainstem lesions and has been demonstrated to have the depth of field penetration up to 30–40 mm, suitable for deeply located tumors [27]. Fortunately, IMT fields have very similar properties to the TTF low-intensity fields produced by external arrays, at 1–3 v/cm and frequencies between 100–300 kHz; therefore, it is reasonable to theorize that TTF will produce similar effects on DIPG cells.

In addition to the additive effects of TTF noted in combination with chemotherapy and radiation, TTF may also play an important role as an adjuvant treatment in immunotherapy trials by stabilizing tumor progression, thereby allowing patients to mount an immune response [6,14]. This is particularly relevant given the recent flurry of exciting results in the immunotherapy sphere for DIPG [6,7,8,25]. TTF are capable of stimulating antitumor immunity properties, which may further produce a synergistic effect in combination with immunotherapies [25]. As such, the combination of TTF with experimental treatment options may represent an exciting new avenue worthy of continued investigation.

In this study, we propose the endoscopic endonasal implantation of a flexible TTF-generating electrode array in the clivus as a potential novel treatment method for DIPG. Extended endoscopic endonasal approaches (EEA) have been extensively described in the literature. The anatomical limits of the transclival approaches have been rigorously studied in adults and are largely defined by the intercarotid distance at the level of the petrous apices as severe bleeding from a potential internal carotid artery injury represents one of the most serious risks [28]. The sixth nerve, which runs along the clivus up towards the cavernous sinus from its exit point in the pons, can also be a limiting factor in extended EEA. When it comes to the pediatric population, EEAs are not as well understood due to the dynamic and variable anatomy within this cohort. Therefore, assessing the size restrictions related to a planned clival implant in this specific age group of patients was necessary. The patients in our study ranged from ages 4–15, with ~60% falling between the ages of 4–7. Sphenoid pneumatization begins between the ages of 2–4 and is largely complete by age 12, although it is highly variable between individuals and can continue well into adulthood [29,30]. Our study confirmed that sphenoid pneumatization, clival length, and the width of the clivus at the sellar floor are significantly correlated with age. In our study population, the average maximum clival width was 1.62 cm. Given these findings, we expect that younger patients may require more extensive drilling to place the implant at the optimal location on the face of the clivus with minimal intervening bone due to lower rates of pneumatization and thicker clival widths. Neuronavigation and Doppler ultrasound will, therefore, be critical tools to safely guide the procedure. The use of intraoperative CT would also be a useful resource to safely guide clival drilling [31]. Recently, augmented reality has also emerged as an adjunct technology that may also improve safe drilling practices, for example, by identifying and localizing the internal carotid arteries and overlaying this patient’s anatomy in the endoscopic field of view [32]. Of note, the intercarotid distance at the level of the clivus did not significantly correlate with age in our population, which is congruent with the literature [33]. Our study population had an average intercarotid distance of 1.68 cm, which would allow the safe implantation of an electrode array with a width of 1.5 cm in most patients.

Accessibility and sufficiency of the working corridor is another concern of pediatric endoscopic surgery, starting from the size of the nostril and including the size of the intranasal cavities. Extensive data has now been published on the feasibility and safety of EE surgery in younger patients, mostly driven by the management of craniopharyngioma [34,35]. Most authors agree on a 3-year age limit for EEA, but a thorough case-by-case evaluation is necessary given significant patient variability. Finally, the vomer–clivus distance, unlike many of the other skull base dimensions, is not dependent on sphenoid pneumatization and does not change significantly during development, and therefore, should not pose an increased limitation in approach in children. Reconstruction is an additional consideration in pediatric EE surgery, as the nasoseptal flap, commonly used for reconstructions in this region, is insufficiently mature for transclival approaches [29,34,35,36]. However, given that our proposed procedure is entirely extradural, reconstruction should not be necessary in most cases. Preserving a thin layer of bone to protect the dura from inadvertent injury may represent the best strategy to avoid accidental durotomies.

Overall, our results confirm that the implantation of a transducer array measuring 3 × 1.5 cm is feasible in 65% of patients due to limitations in either clival length or intercarotid distance, and that a 2.5 × 1 cm array would be implantable in 94% of patients studied. These proportions are within the dimensions of currently available clinical systems produced by Optune (Novocure, Haifa, Israel), which offers a ceramic array measuring ~ 2 cm. An important consideration in the design of the transducer array will be the ability to easily remove and replace the device, given that it is expected that this population of patients will require multiple MRIs over the course of their management. We propose a detachable wiring system to allow the removal of the non-MRI compatible portion prior to scans. Such a procedure could likely be performed as an outpatient with minimal anesthesia, or under anesthesia concomitantly with a sedated MRI. We anticipate that array wiring exiting through the nasal cavity can be managed similarly to a nasogastric tube when the device is in use (Figure 2).

**Figure 3:**
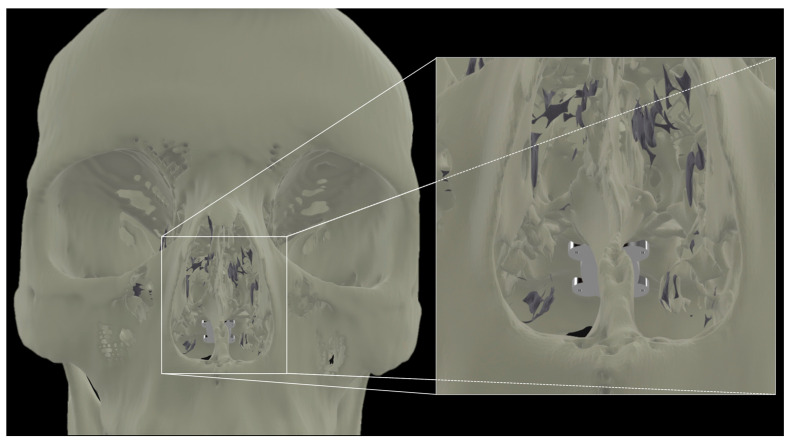
Transnasal view of proposed placement of a flexible array affixed to the clivus.

Our report confirms the anatomical feasibility of the transclival approach for TTF enhancement. The next steps include the finite element modeling of the electrode array configuration for optimal pontine coverage. In a recently published study, we demonstrated the potential efficacy of intracranial electrode arrays [26]. In doing so, we have defined configurations that universally required less current while still reaching significantly higher field strengths and therapeutic enhancement ratios (TER) in larger portions of the tumor bed as compared to transcranial controls. Some groups have posited that the optimal transcranial array placement to achieve sufficient coverage in the brainstem would involve the placement of arrays on the vertex, bilateral posterolateral occiput, and superior-posterior neck [37]. A transclival array may achieve adequate electric field distribution with minimal transcranial complementary arrays and less amperage, enhancing the practicality of the long-term use of the TTFs. Another consideration and active area of research is the material engineering of a flexible array that will permit insertion through the nose and fixation to the clival bone (Figure 3).

To advance this work, a model device will be employed in an effort to demonstrate the safety of implantation, tolerability, and biocompatibility in a large animal model. Sheep have commonly been used in endoscopic endonasal models due to the relative anatomic similarities of the nasal cavity and sinus orientation between sheep and humans [38,39]. The ultimate goal of this study is to bring this device and approach to the clinic as a novel potential treatment method for DIPG.

## 5. Limitations

This work represents the first step towards assessing the feasibility of transclival TTF electrode implantation in a pediatric cohort. Only patients who had a brain CT scan were included in our analyses, which limited our cohort to 17 patients, as MRIs are the preferred test for assessing brain tumors. The authors acknowledge that this sample size is limited; however, we believe it is sufficient to achieve our aim of demonstrating the feasibility of such an approach in the average pediatric patient. Tumor field modeling is currently ongoing to assess the optimal placement of complementary array(s). In vitro evaluation and preclinical animal models are also necessary to establish efficacy and advance this work from the bench to the bedside. In addition, the array will need to be engineered with the ability to install and remove it with relative ease to facilitate MR imaging. In this setting, the evaluation of long-term biocompatibility and clinical tolerance/uptake will also be necessary.

## 6. Conclusions

Endoscopic endonasal transclival implantation of a TTF array is anatomically feasible in the vast majority of pediatric DIPG patients assessed, and as such, may represent a novel adjuvant treatment for this intractable disease.

## 7. Patents

There were 63/345,878 patients filed on 25 May 2022 and 63/400,002 filed on 22 August 2022.

## Figures and Tables

**Table 1:** Demographics.

Sex	
Male	10
Female	7
Age	
4–7	10
8–11	5
12–15	2
Sphenoid pneumatization	
Conchal	6
Presellar	5
Sellar	4
Postsellar	1

**Table 2:** Anatomical measurements.

	Average	Standard Deviation
Clival length (floor of sella to basion)	3.34	0.39
Width of clivus at sellar floor	1.43	0.69
Greatest width of clivus	1.62	0.19
Intercarotid distance	1.68	0.36

**Table 3:** Relationship between age and anatomical measurements.

	R^2^	*p*-Value
Clival length (floor of sella to basion)	0.568	0.0005 *
Width of clivus at sellar floor	0.372	0.009 *
Greatest width of clivus	0.004	0.82
Intercarotid distance	0.006	0.76
Sphenoid pneumatization	0.605	0.0002 *

* indicates significance *p* ≥ 0.05.

## Data Availability

Not applicable.
